# Mite non-reproduction, recapping behavior, and hygienic behavior (freeze-kill method) linked to *Varroa destructor* infestation levels in selected *Apis mellifera* colonies

**DOI:** 10.1177/10406387231172141

**Published:** 2023-05-04

**Authors:** Marie-Lou Morin, Pierre Giovenazzo

**Affiliations:** Département de biologie, Université Laval, Québec City, Québec, Canada; Département de biologie, Université Laval, Québec City, Québec, Canada

**Keywords:** *Apis mellifera*, honey bees, hygienic behavior, mite non-reproduction, recapping, *Varroa destructor*

## Abstract

The genetic selection of honey bees (*Apis mellifera*) possessing specific social hygienic behaviors offers the beekeeping industry the possibility of controlling the *Varroa destructor* parasite and thus reducing its dependence on acaricides. However, the links between these behavioral traits are not yet well defined, which limits genetic progress in breeding programs. We measured the following behavioral varroa resistance traits: freeze-kill brood (FKB) and pin-kill brood (PKB) assays, varroa-sensitive hygiene (VSH), pupae removal, mite non-reproduction (MNR), and recapping activity. We found 2 negative and significant relationships: 1) between the recapping of cells infested with varroa and the total number of recapped cells, and 2) between the recapping of cells infested with varroa and VSH. We also selected the best predictive model of varroa infestation levels using the “step-wise” approach based on the Akaike information criterion. Our model revealed that MNR and FKB were significantly related to the varroa population levels, with a negative relationship; recapping was significantly related to mite infestation levels, with a positive relationship. Thus, a higher MNR or FKB score was linked to lower levels of mite infestation in colonies on August 14 (prior to fall infestation treatments); a higher recapping activity was linked to a higher level of mite infestation. Recapping behavior could be a useful trait to aid the selection of varroa-resistant bee lineages.

*Varroa destructor*^1^ is an ectoparasite of the honey bee (*Apis mellifera*) and represents the greatest biologic threat to apiculture globally,^48,58,59^ causing significant losses in colonies and thus affecting beekeeping profitability. The parasite is completely dependent on its host, feeding on the fatty body tissues and hemolymph of both adult bees and brood,^56^ and using the bee’s metamorphosis stage in capped brood to reproduce.

Various approaches are used by beekeepers to reduce varroa mite infestation in their colonies. The treatment recommended most commonly is the use of medicated strips coated with synthetic acaricides. Unfortunately, resistance to these products in populations of varroa mites has appeared worldwide,^35,42,59^ and acaricide residues have been found to contaminate honey bee products.^6,66^ In addition, the effect of these pesticides has repercussions on the health of the host bees.^57,65^

An alternative strategy, being developed within breeding programs based on quantitative genetics,^39^ is the selection of heritable traits that provide resistance or tolerance to varroa. *Resistance* allows colonies to survive by reducing the growth of the varroa population; *tolerance* reduces parasite impacts despite uncontrolled levels of infestation.^60^ Several traits that confer resistance have been observed in natural populations of *A. mellifera* surviving with *V. destructor* without external intervention.^24,32,33,51,61^ The traits of interest for genetic selection are primarily behavioral defenses, part of the social immunity of bees. “Social immune systems result from the cooperation of the individual group members to combat the increased risk of disease transmission that arises from sociality and group living.”^14^ Indeed, the proximity of individuals in the hive makes the colony vulnerable to infections and parasites.

Certain populations of honey bees exhibit so-called *hygienic behaviors*, which means that they detect and remove dead or diseased brood from the colony to limit the spread of pathogens to healthy individuals. Studies have demonstrated the effectiveness of hygienic behavior in resisting the bacterium *Paenibacillus larvae*, the cause of American foulbrood,^63^ and the fungus *Ascosphaera apis*, which is responsible for chalkbrood disease.^19^ Typically, this trait is artificially selected by estimating the hygiene level of a colony based on tests designed to kill a known amount of capped brood and then checking the percentage of pupae removed after a pre-established time has elapsed. In North America, the test used is the freeze-kill brood (FKB) assay (or freeze-kill brood test), in which liquid nitrogen is used to kill the brood.^9^ In the pin-kill brood (PKB) assay (or pin-test), used for the same purpose in Europe, pupae are killed with a needle that pierces through the wax cell cap and cocoon.^9^ Compared to the FKB assay, the PKB assay typically results in higher hygienic levels, because the response is faster given the hole created in the cell cap^53^ and the leaking of pupal fluids from the perforation.^20^ Although the colony benefits from considerable immunity as a result of such hygienic behavior,^8,10^ these tests likely do not identify varroa resistance. In fact, bees detect varroa mites and dead brood by different stimuli.^41,45,47,68^ Furthermore, measuring hygienic behavior with the FKB assay has shown that it is a poor predictor of colony varroa infestation level,^31^ and selection of this trait alone has not conferred resistance to the parasite.^64^ However, colonies with hygienic behavior levels measured by the PKB assay had significantly lower mite infestation levels than non-hygienic colonies,^18,62^ and had lower load or prevalence of viruses.^18^

Approximately 2 decades ago, colonies capable of a form of hygienic behavior that is specific to *V. destructor*, referred to as varroa-sensitive hygiene behavior (VSH), were identified.^23,28,29^ VSH has thus become an important focus of studies on varroa resistance.^25^ This trait manifests itself in a way similar to typical hygienic behavior, but workers detect and remove the brood infested by a reproducing varroa mite and not a dead pupa.^23,27^ This behavior reduces varroa infestation levels in the brood^7,15–17,24,30,38^ and has an apparent link to varroa resistance in multiple populations.^3,4,54^ Despite this, the significance of this trait in human-assisted selection programs is questionable, given that only one instance of VSH’s heritability measurement has been reported,^8^ and the value was rather low (0.18). Furthermore, testing for this trait is very demanding in terms of both time and resources because it requires tending to highly infested colonies as varroa reservoirs. The logistical implications of testing for this trait are an important hindrance to its inclusion in breeding programs, in which hundreds of colonies must be tested to achieve genetic progress.

Another trait associated with adult workers, and an interesting resistance mechanism, is recapping behavior. Bees that engage in recapping behavior detect mites that are in the process of reproducing in a brood cell and partially open the wax cover, then close it again with fresh wax. This behavior is thought to disturb the parasite during its reproductive cycle, thereby reducing its fertility,^49^ although other studies found that varroa reproduction was not impacted by natural recapping activity.^26,37^ Furthermore, the recapping trait was reported to have low repeatability and heritability with no link to mite infestation levels.^5,22^

There are also traits innate to the brood, such as accelerated death or entombment of pupae, which increase brood removal by workers that react to the release of odorant molecules.^43,45,49,52^ The effect on varroa is the same: all of these traits prevent varroa mites from reproducing properly. This phenomenon, formerly called suppressed mite reproduction (SMR),^24^ was recently renamed *mite non-reproduction* (MNR),^44^ to restrict the definition of the term SMR to those cases in which varroa reproduction is affected by brood exclusively. Many factors can cause MNR, from attributes of the varroa mite itself (fecundity and fertility) to attributes of the *A. mellifera* workers or brood. MNR can be detected by investigating capped brood cells and identifying abnormalities in the offspring produced by the mite.^12^ This resistance trait interferes with the reproductive cycle of mother mites (foundresses) and thus lowers the infestation level continuously throughout the season.

Comparative phenotypic analyses of resistant colonies seem to indicate that there is no universal mechanism providing resistance.^44^ In fact, the great variation in traits^33^ present in surviving colonies suggests that effective resistance is not the result of a single trait but rather a combination of traits.^44^ Furthermore, although selection programs have been working toward increasing the frequency of behavioral resistance traits since the 1980s,^11^ survival of untreated commercial colonies has yet to be achieved.^50^ Indeed, beekeeping management significantly affects infestation levels and defense behavior, given that drift and robbing behavior cause horizontal transmission of varroa between colonies.^21^ More studies are needed to establish the links between the different traits and defining proxies to be able to perform artificial selection assisted by molecular markers. Such a breakthrough would undoubtedly accelerate the genetic progress of varroa resistance because these methods are faster and more cost-effective than current phenotypic assessment methods.

Our main objective was therefore to improve knowledge of the links between behavioral traits that contribute to varroa resistance and social immunity in colonies. The specific objectives were: 1) to measure the correlations between the FKB assay, the PKB assay, MNR, VSH, varroa-infested pupae removal, pupae recapping activity, and colony varroa infestation levels; and 2) to identify which of the above traits best predict colony varroa infestation levels on August 14 prior to fall infestation treatments.

## Materials and methods

### Colony establishment and management

In early May 2019, 56 colonies of similar strength (total brood area) and comprising 4 groups of sister queens (*n* = 14 per group) were chosen among the bee stock that has been selectively bred for hygienic behavior, as measured with the FKB assay, for > 10 y at our bee research facility, the Centre de Recherche en sciences animales de Deschambault (CRSAD; Québec, Canada). Young sister queens were introduced in colonies in July 2018. Colonies of each group were randomly and equally distributed on 5 different apiary sites selected to represent the standard agricultural profile of farmland in the region surrounding our research facility (71° 39' 49.966'' N; 46° 45' 25.129'' O). Drifting between colonies was minimized by randomizing the orientation of the hive flight opening. Colonies were managed with double brood chambers in 10-frame Langstroth boxes and inspected biweekly throughout 2019; honey supers were added as needed. During these colony inspections, every sign of disease was recorded, and all swarm cells were removed. All colonies that had either died (or whose queen had died), or that showed visible signs of European foulbrood or chalkbrood, were removed from the project. We maintained 34 colonies until the end of the experiment and included them in the data analyzed.

### Hygienic testing: FKB and PKB assays

Hygienic testing was carried out twice, outside of honey flow (July 23–26 and August 5–9, 2019). The FKB and PKB assays were carried out on the same brood frame at the same time. For each repetition, patches of worker brood at the pupal stage (pink-to-purple eyes) were killed to check the hygiene level.^9^ Briefly, for the FKB assay, 100 mL of liquid nitrogen was poured on 2 circular areas (15-cm diameter) within the brood area of each hive. The liquid nitrogen was confined to a specific spot on the brood frame (inserted section of a PVC tube) and covered an area of 120 cells. For the PKB assay, a rhomboid stencil of 10 × 10 cells (surface of 100 cells) was used to pierce 2 patches of 50 cells with a disinfected sewing pin, for a total of 100 cells per colony. Frames that included the tests performed were put back in the center of the top brood chamber. Hygienic behavior was evaluated by calculating the number of frozen or pin-killed pupae completely removed after 9 h for PKB and after 24 h for FKB, expressed as a percentage.

### VSH, removal of pupae, MNR, and recapping activity

#### Preparation of the varroa-infested brood frame

**Figure 1.**
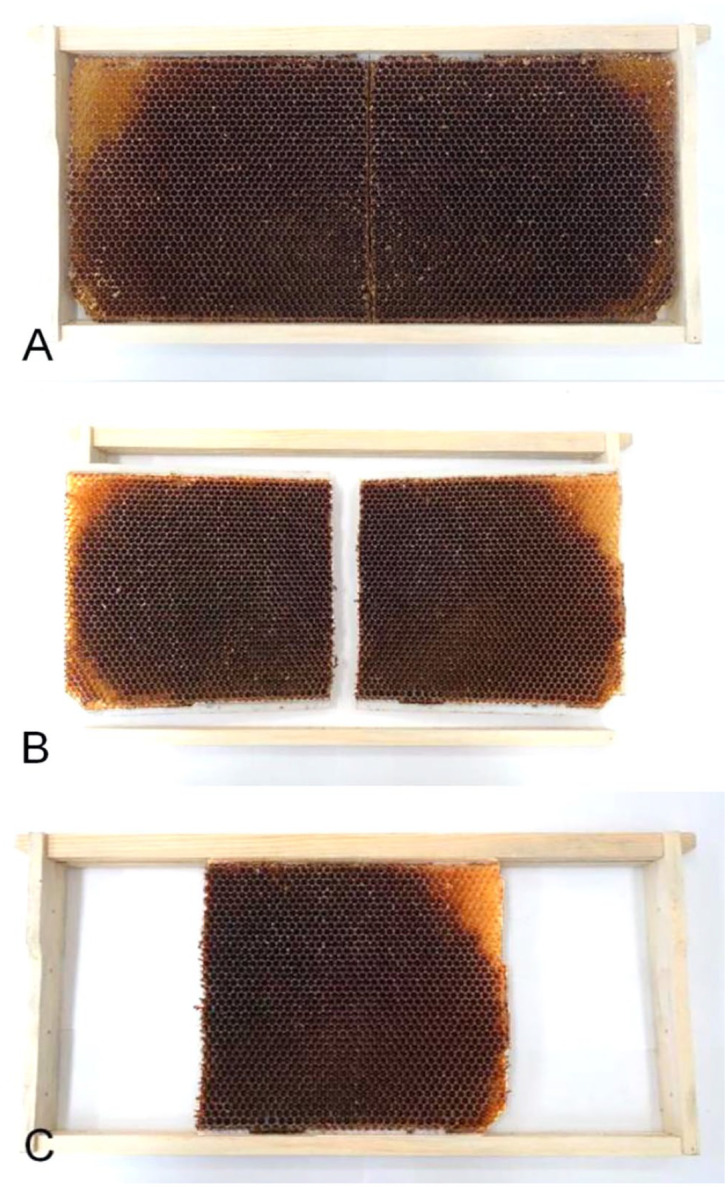
Modified frames used for testing varroa-sensitive hygiene, pupae removal, mite non-reproduction, and recapping activity. This frame was placed in a varroa-infested colony, then each section was placed in a testing colony. **A.** Langstroth frame with foundation cut in half. **B.** Langstroth frame taken apart, foundation cut in half. **C.** Half of foundation mounted in frame. (Photos by S. Rouleau-Breton).

The honey bee queen of a varroa-infested colony was captured and placed in a single-frame queen excluder cage with a modified brood frame separated into 2 sections (Fig. 1A). This procedure was developed to reduce the number of donor colonies. This caged frame with the queen was returned to its colony for 48 h for the queen to lay her eggs. After 48 h, the queen was released and the frame was left in the colony, leaving varroa infestation to occur freely until capping of cells (9–10 d). The inoculated capped brood frame was then removed from the infested colony and separated in two, and each half (Fig. 1B, 1C) was then reattached in an empty frame. Initial varroa infestation rates were estimated in each half frame by inspecting 150 random brood cells, 75 cells per side. If the infestation rate was > 10%, the half frame was identified and assigned to a test colony. Photos were taken of each side of a half frame for brood pattern reference. Once all frames had been surveyed, they were placed in the center of the brood box of a test colony. Half frames were left undisturbed for 7 d,^12^ were photographed again, and frozen at −20°C until evaluations were made of VSH and MNR.^12^

#### Varroa-sensitive hygiene

VSH was estimated by measuring the change in varroa infestation of capped brood before and after incubating in colonies, in 150 cells each time. VSH level of a colony was evaluated by calculating the difference between the initial and final varroa infestation rates of the inoculated frame expressed as a percentage of the initial infestation rate of the inoculated frame.^67^

#### Removal rate of pupae

The number of pupae in capped brood removed from inoculated frames was counted by comparing photographs taken before and after incubating in colonies. Removal rate of pupae for each colony was evaluated by calculating the number of pupae removed from capped brood from the final photo expressed as a percentage of the initial total brood cells in the initial photo.

#### Mite non-reproduction

Reproduction failure of varroa mites within brood cells was measured.^12^ Briefly, brood cells from the inoculated frames were carefully uncapped, and the presence of mites was investigated. In each infested cell, the family of mites present was described. In a 9-d post-capped cell, a foundress varroa is normally accompanied by at least 1 young adult female and 1 adult male. When this was the case, the mite was deemed reproductive (R). If an infested cell did not contain this offspring, the mite was considered non-reproductive (MNR). Cells from a frame were randomly verified until 35 infested cells were obtained, or until 700 cells were opened. MNR rate of a colony was evaluated by calculating the number of non-reproducing mites expressed as a percentage of the total infested cells investigated.

#### Recapping activity

To evaluate this behavior, the inner surface of each cell capping was examined. If no manipulation had been performed by the bees, the silk cocoon remained intact. However, the presence of a matte hole on the inner surface indicates that it had been uncapped and recapped by the bees. Recapping rate of a colony was evaluated by calculating the number of recapped cells expressed as a percentage of the total cells verified. For additional insight into recapping behavior, we also compared the total recapping rate (as calculated above) to the recapping rate of infested cells only. This indicates whether recapping behavior targets infested cells specifically or if the behavior is random.

#### Varroa infestation rate in colonies

The initial and final mite population in each colony (May 2019 and August 2019) was evaluated using a standard ethanol wash method.^9^ Briefly, ~300 bees were removed from a brood frame and euthanized in a 70% ethanol solution. Bee samples were shaken, and fallen mites were counted. A subsample of 50 bees was weighed, as was the whole sample, to calculate the exact number of bees in each sample using simple linear regression. Varroa infestation level was evaluated by calculating the total number of mites expressed as a percentage of the number of bees in the sample. All but 3 colonies had an initial infestation estimate of 0%; hence, we used only the August infestation rate.

#### Statistical analyses

Correlations between variables were verified using the Spearman rank-order coefficient, and *p*-values were Bonferroni corrected. A Poisson generalized linear mixed model was used to study the effect of the different hygienic behaviors (FKB, PKB, VSH, removal rates, MNR, and recapping rates) on the rates of colony infestation by *V. destructor* in August. In our model, the response variable is the number of infested bees per colony, which is linked to the explanatory variables with a log link function. Moreover, the log of the total honey bees in varroa samples was used as an offset variable, and the 5 apiary sites were integrated in our model as a random effect. The most important explanatory variables were selected using the stepwise approach based on the Akaike information criterion (AIC). For this, the likelihood function was approximated using the adaptative Gauss–Hermite quadrature with k = 5 points. Furthermore, collinearity between variables was verified using the variance inflation function (VIF). All analyses were done in R using the lme() function of the nlme package (R v.4.0.4, https://svn.r-project.org/R-packages/trunk/nlme/).

## Results

### Colony-level phenotypes

The 2 hygienic tests resulted in different average hygienic percentages. The FKB tests had an average of 88.0% removal of dead pupae, and the PKB tests yielded a lower average of 52.9% (Table 1). Average values for VSH and MNR were 45.7% and 31.4%, respectively. Nearly half of all brood cells were recapped by honeybees (43.4%), and 30.3% of these recapped cells were varroa-infested. Average varroa infestation rates were 0.6% on August 14.

**Table 1.** Statistical characteristics of variables measured in tested honey bee colonies (*n* = 34).

Variable	x̄, %	SE	Min.	Max.
Hygienic testing				
Freeze-kill brood assay	88.0	1.9	51.3	100.0
Pin-kill brood assay	52.9	2.7	20.5	77.0
Varroa-sensitive hygiene	45.7	4.2	0	88.9
Pupae removal	15.1	1.2	2.5	38.0
Mite non-reproduction	31.4	1.8	12.8	60.5
Recapping activity				
Total cells recapped	43.4	3.5	15.5	95.7
Recapped cells infested by varroa	30.3	2.5	6.1	57.6
Varroa infestation rate	0.6	0.1	0	2.6

Max. = maximum; Min. = minimum.

**Figure 2.**
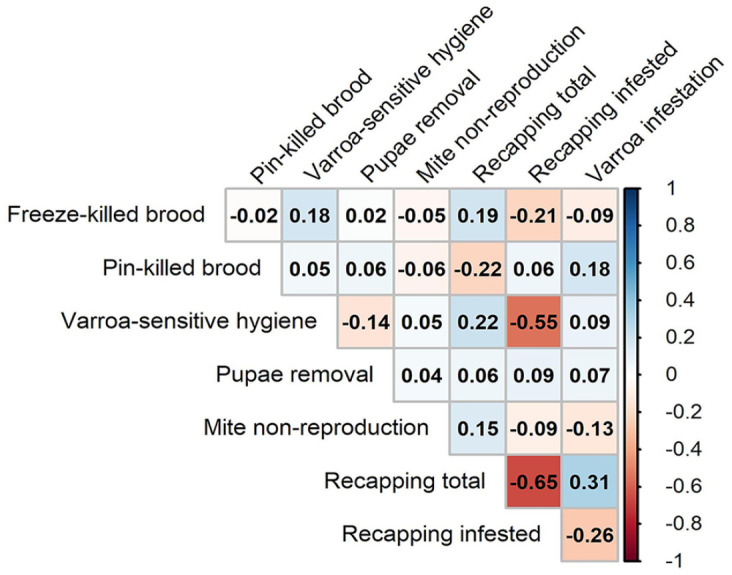
Varroa-related colony phenotypes correlation matrix using the Spearman rank-order coefficient statistic. Correlation coefficients (reported as ρ values) are shown for each pairwise comparison. Shaded blue cells represent positive correlations, and red cells represent negative correlations. Darker hues indicate stronger correlations as indicated by the correlation color gradient on the right.

To explore the dynamics of the various varroa-related traits, we calculated the correlations between each pair of variables (Fig. 2). Of the 28 pairs of variables, 2 were significantly correlated after a Bonferroni correction (α = 0.0018). The proportion of recapping among mite-infested cells (recapped cells and varroa-infested) was negatively correlated with the proportion of recapping among all cells (total cells recapped; ρ = −0.65, *p* < 0.0001) and was also negatively correlated with VSH (ρ = −0.55, *p* = 0.0008). The proportion of recapping among all cells was positively correlated with mite infestation levels (varroas), although the result was only marginally significant (ρ = 0.31, *p* = 0.073). Because ρ-values of the correlations are below |0.7|, they are considered weak relationships.

### Predicting mite infestation level

**Figure 3.**
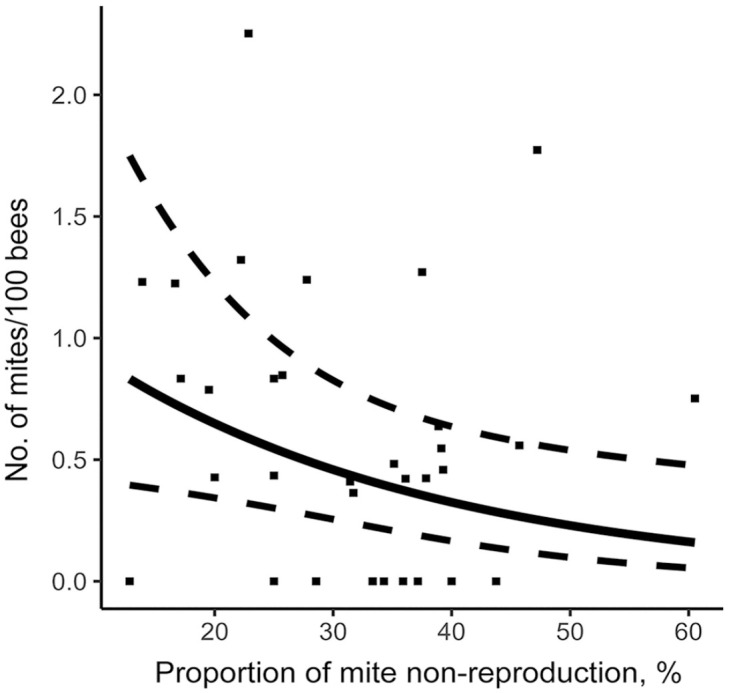
Relationship between mite non-reproduction and mite infestation level in colonies on August 14. Prediction model is represented by a full line, 95% CIs by dotted lines, and original data of each colony by points.

**Figure 4.**
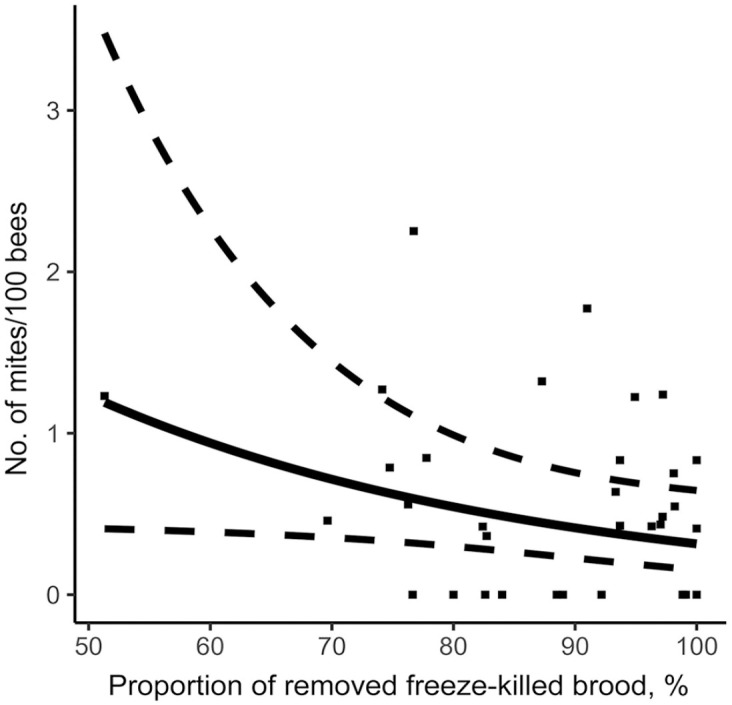
Relationship between freeze-kill brood test and mite infestation level in colonies on August 14. Prediction model is represented by a full line, 95% CIs by dotted lines, and original data of each colony by points.

**Figure 5.**
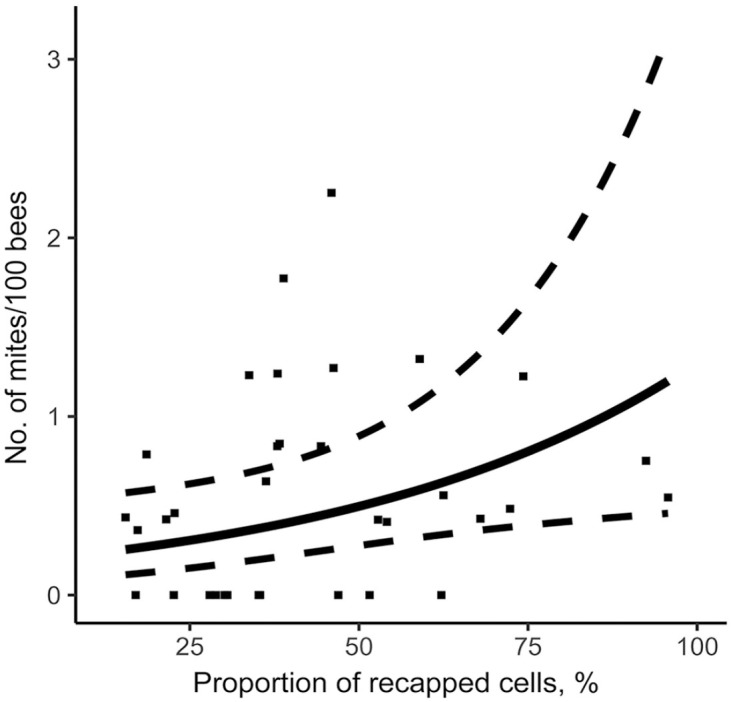
Relationship between total recapping activity and mite infestation level in colonies on August 14. Prediction model is represented by a full line, 95% CIs by dotted lines, and original data of each colony by points.

Using the stepwise approach based on the AIC criterion, our model measured the effect of the various hygienic traits measured on the *V. destructor* infestation rate of colonies in August (Table 2). MNR and FKB were significantly negatively linked to varroa population. Thus, a higher MNR score (Fig. 3) or FKB score (Fig. 4) is a significant predictor of a low colony mite infestation level in August. Furthermore, our model showed that higher recapping activity is significantly positively linked to higher mite infestation levels. Thus, higher recapping activity (Fig. 5) is a significant predictor of a high colony mite infestation level in August. In comparison, the most important explanatory variable using the stepwise approach is MNR (AIC = 53.6), followed by the total recapping activity (AIC = 52.4) and FKB (AIC = 50.4; Table 2). These predictions are valid within the value ranges of each variable (Table 1). However, the SEs are rather large compared to the estimate (Table 2). Thus, although the factors are significant in the model, the infestation rates of some colonies do not fall within the 95% CIs of the model (Figs. 3, 5).

**Table 2.** Estimates, SEs, *z*-values, and *p*-values of fixed-effect variables in a general linear model of mixed effects for Poisson regression (glmer).

Variable	Estimate	SE	*z*	*p*
Intercept	−2.778	1.085	−2.561	0.010
Mite non-reproduction	−3.460	1.476	−2.345	0.019
Total cells recapped	1.929	0.843	2.287	0.022
Freeze-kill brood assay	−2.731	1.378	−1.982	0.047

Random effect = apiary; offset variable = log of the total honeybees in samples for varroa counts; *n* = 34 colonies.

## Discussion

Three of the traits studied were significant in our predictive model of colony phoretic mite infestation level: MNR, recapping activity, and hygienic behavior as measured with the FKB assay. Higher levels of MNR or FKB predicted a lower mite infestation level in the colony; a high recapping activity of all cells from a brood frame predicted a higher varroa infestation level in the colony. The other traits (PKB, VSH, removal of pupae, and recapping of infested cells only) did not significantly improve the prediction of the mite infestation level within a colony.

Our findings, while interesting, have limitations. A study^46^ showed that MNR scores vary considerably with the number of cells investigated, as demonstrated by using the cumulative density functions of the MNR estimate. For instance, investigating 10 single-infested cells (SIC) showed a raw variability of ± 30%, investigating 35 SIC a variability of ± 20%, and 100 SIC, ± 12%. Our sampling numbers were based on previous studies of SMR, in which observations typically varied between 30 and 50 single-infested cells^9,34^ and thus may have reduced the power of our analysis. By using naturally infested brood frames for our tests, high infestation rates were not always present. Investigating frames for 35 single-infested cells (or more) is time-consuming when the varroa infestation level is < 30% (100 random cells must be investigated to reach 30 infested cells). One solution to improve the method would be to artificially infest a brood frame with varroa collected from infested colonies, as has been done by other researchers.^17,36,46^ This would also ensure equal infestation rates for all colonies, but we suspect that manipulation of the capped brood could have an impact on olfactory cues and cause some bias of honey bee hygienic behavior.

The significance of the recapping activity in selecting for varroa resistance is unclear in the literature.^44,66^ Previous studies suggested that recapping activity is linked to varroa resistance, given that the worker bees detect infested capped brood and disrupt the reproducing foundress by uncapping the cells. This is thought to affect cell humidity and temperature, with a possibly fatal impact on the young varroa male, and thus have a negative impact on the mite’s reproduction. This phenomenon was observed when brood cells were uncapped and recapped experimentally.^51^ If this were the case, we should have observed a positive correlation between recapping and MNR, the 2 measures predicting a lower mite infestation level, but we did not. Although such a correlation has been demonstrated in experiments, it has been disproven in naturally surviving populations.^26,37,51^ Our results suggest that recapping activity does not indicate resistance towards varroa, but rather that worker recapping activity increases in colonies with a higher level of infestation (Fig. 4). It may also show the inefficiency of this hygienic behavior, or that this behavior is not yet well adapted within *A. mellifera*. This adds to the results of studies that found a mismatch between high recapping rates and VSH,^17^ or hygienic behavior.^2^ The differential results obtained could also mean that temperature, humidity, or other unidentified factors, do not have the same impact on MNR in all populations.^21^ Indeed, the survival and reproductive success of the mite after it leaves an uncapped cell is variable between studies,^13^ which could in turn affect the colony’s resistance to the parasite. This, combined with the absence of convincing data on the trait’s heritability, suggests that recapping behavior plasticity may be high, and dependent on developmental factors, rather than purely genetics. Given that we observed higher recapping rates in the more infested colonies, it is possible that the olfactory triggers were more abundant in those colonies, resulting in a stronger behavior, and not that the behavior impacts infestation levels. Nevertheless, there was a negative correlation between recapping rates of infested cells, which suggests that bees recapped fewer infested cells.

The other significant correlation that we found was a moderate negative relationship between recapping rates of infested cells and VSH levels. VSH is the removal of infested pupae, which results in a reduction of net varroa infestation rate in brood. The recapping behavior can be seen as the first step in VSH behavior, the uncapping of the infested cell. However, rather than removing the pupa and mite, the cell is recapped, leaving both the pupa and mite in the cell. Thus, if the workers of a colony recap most of the uncapped infested cells, VSH should occur in lower frequency, based on our results. On the other hand, VSH was expected to be positively correlated with the number of pupae removed from the frame during the test, as measured by photographs taken before and after insertion into the hive. Workers that express VSH behavior decrease varroa infestation in the brood by removing infested pupae. In noting a lack of significant correlation, we believe that the number of pupae removed is dependent on factors other than VSH behavior, such as other diseases or environmental factors.

Hygienic behavior appraised by the FKB assay is an indication of the ability of a colony to remove dead (frozen) brood from the nest quickly. This would explain why hygienic behavior has never been strongly associated with varroa resistance. However, our results suggest that there is a link between FKB and varroa infestation levels, given that the FKB score was a significant predictor of a low mite infestation level in a colony. This is surprising because it contradicts the findings of others^55^ that PKB assays rather than FKB assays predicted varroa non-reproduction in single drone-inseminated colonies. These contradictory results could be explained by the fact that our bee stock has been selectively bred for hygienic behavior as measured with the FKB assay for > 10 y. We know that the trait is heritable in CRSAD’s stock^39^ and that genetic progress for this trait has been confirmed.^40^ Furthermore, other researchers^55^ found a lack of correlation between FKB and PKB just as we did, likely as a result of a different heritable mechanism at play in the observed behavior.

Although FKB was found to predict varroa loads in August, our model’s predictive capability is weak, given that many plot points were outside the 95% CIs. Also, we found no correlations with other variables to be significant. The most surprising result is the absence of correlation with PKB because both tests are linked to the same behavior. Major differences in the methods could explain this discrepancy, as well as the argument previously discussed. FKB seems to kill all pupae more reliably during the test. With PKB, manual piercing of each pupa increases the chances of only injuring them. If pupae are not all killed, hygienic behavior can be impacted. Furthermore, perforation of the cell cap and the subsequent deposit of pupal fluid on the frames could also affect worker behavior. One must therefore be cautious when comparing the hygiene levels of bee populations determined by different tests. FKB is a reliable test for selecting hygienic behavior, and our results show that it is linked in some way to varroa infestation levels.

Overall, our results show that MNR and FKB could potentially be used as indicators of varroa resistance in honey bee colonies because we observed that these traits significantly predict lower infestation rates in August. Recapping, on the other hand, is an indicator of higher infestation levels. However, it will be important for future research to validate these results on unselected colonies and demonstrate the heritability of *V. destructor* resistance traits in *A. mellifera* populations. Without this, genetic selection efforts will not yield genetic progress. One avenue that should be considered, despite the risks it entails, is colony selection based on survival, rather than the expression of one or a few preselected behaviors. The fight against *V. destructor* is far from over, and it is imperative that we add several weapons to bees’ defensive arsenal, or the battle will never be won.
